# Twilight Sleep, Is It Applicable to Dentistry?

**Published:** 1915-04

**Authors:** M. L. Rudolph

**Affiliations:** Clarksville, Tenn.


### TWILIGHT SLEEP, IS IT APPLICABLE TO DENTISTRY?

BY M. L. RUDOLPH, D.D.S., CLARKSVILLE, TENN.

A definite answer would depend on a more extensive use of the drug in the profession, but I think worthy of further investigation.

My very limited use has given perfect results. Twilight sleep was suggested to me by the wife of a physician, who had taken it in childbirth with such pleasing results that on applying to me for the extraction of a badly broken-down third molar, she insisted on taking this anesthesia in preference to all others. Her husband said cocaine and its alkaloids was contraindicated and a perfect idiosyncrasy for N2 O & O. Owing to her highly nervous condition some kind of anesthesia was imperative. This anesthesia was given her, and the operation was performed without the least pain or excitement.

The writer believes it could be more advantageously used in operative dentistry, as the patient is always conscious and will give it a trial on first opportunity.

Hoping this will meet the eyes of some one in the profession, who will also investigate along this line.

